# Enhancing the Scalability of Crystallization-Driven Self-Assembly Using Flow Reactors

**DOI:** 10.1021/acsmacrolett.3c00600

**Published:** 2023-11-16

**Authors:** Laihui Xiao, Sam J. Parkinson, Tianlai Xia, Phillippa Edge, Rachel K. O’Reilly

**Affiliations:** School of Chemistry, University of Birmingham, Edgbaston, Birmingham B15 2TT, U.K.

## Abstract

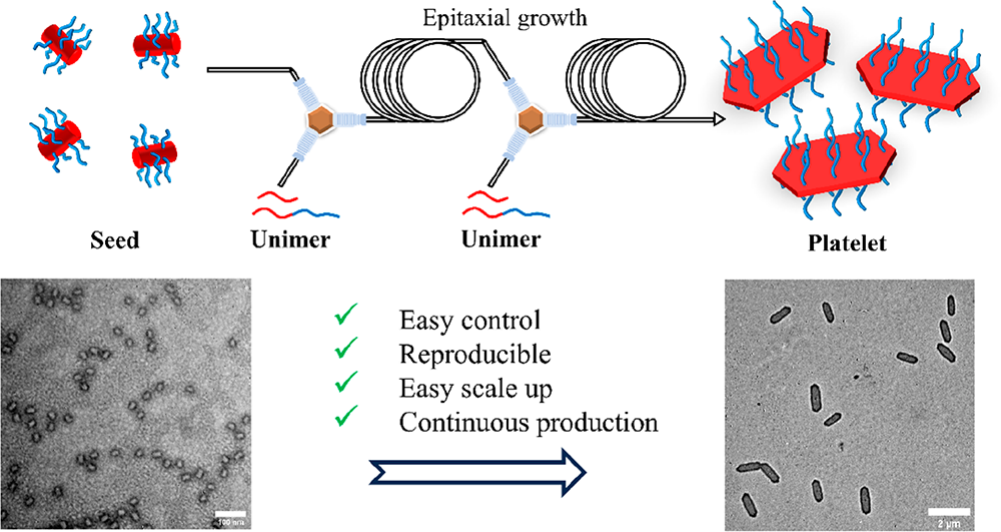

Anisotropic materials have garnered significant attention due to their potential applications in cargo delivery, surface modification, and composite reinforcement. Crystallization-driven self-assembly (CDSA) is a practical way to access anisotropic structures, such as 2D platelets. Living CDSA, where platelets are formed by using seed particles, allows the platelet size to be well controlled. Nonetheless, the current method of platelet preparation is restricted to low concentrations and small scales, resulting in inefficient production, which hampers its potential for commercial applications. To address this limitation, continuous flow reactors were employed to improve the production efficiency. Flow platforms ensure consistent product quality by maintaining the same parameters throughout the process, circumventing batch-to-batch variations and discrepancies observed during scale-up. In this study, we present the first demonstration of living CDSA performed within flow reactors. A continuous flow system was established, and the epitaxial growth of platelets was initially conducted to study the influence of flow parameters such as temperature, residence time, and flow rate on the morphology of platelets. Comparison of different epitaxial growth manners of seeds and platelets was made when using seeds to perform living CDSA. Size-controllable platelets from seeds can be obtained from a series flow system by easily tuning flow rates. Additionally, uniform platelets were continuously collected, exhibiting improved size and dispersity compared to those obtained in batch reactions.

Two-Dimensional (2D) polymeric platelets are gaining great attention because of their diverse range of applications.^1−3^ Specifically, 2D platelets with high aspect ratios have attracted significant interest due to their exceptional properties in cargo delivery,^4,5^ surface modification^6^ and composite reinforcement.^7^ Compared to traditional self-assembly, by which pure platelets cannot be generally obtained, crystallization-driven self-assembly (CDSA) can easily access this morphology. Furthermore, using a seeded growth method, known as living CDSA, precise control over the size of the platelets can be achieved by adjusting the amount of unimer added to the seed solution. Several studies have reported successful preparation of platelets using semicrystalline block copolymers such as polyferrocenylsilane (PFS),^8−10^ polycaprolactone (PCL),^5,11,12^ and polylactide (PLLA).^6,7,13^ While these advancements have made platelet synthesis more accessible, scaling up the production remains challenging, as increasing the scale means slower diffusion of unimer and due to the rapid rate of crystallization this leads to an increase in size dispersity. Thus, the rapid crystallization rate associated with living CDSA limits its application to small-scale and dilute solutions (<1 wt % solids).^14,15^ Undoubtedly, this limitation hinders its potential for widespread commercial use.

Flow chemistry, where reactions are carried out in a continuous stream, is seeing significant uptake because of its universality for chemistry.^16,17^ Flow reactors provide advantages in terms of efficiency, safety, and scalability.^18,19^ Batch scale-up often suffers from poor reproducibility due to inadequate heat transfer and mixing profiles, leading to reaction inhomogeneity, lack of control, and potential hazards.^17,18^ In contrast, flow reactors, with their high surface area to volume ratio offer improved heat transfer, maintaining an isothermal reaction environment even at high temperatures, thus promoting homogeneous and rapid reactions in a safe manner.^18,20−23^

A variety of polymer self-assembly techniques has been transferred to flow platforms. For instance, Junkers and co-workers conducted solvent-driven self-assembly of amphiphilic block copolymers in flow. They found that using turbulent mixing to homogenize the solution yielded more uniform and reproducible flow micelles compared to batch production. Furthermore, the aspect ratio of micelles was highly influenced by the flow rate.^24^ Wang et al. illustrated that the shear forces in flow altered the morphologies of nanoparticles from those observed in batch, allowing more complex kinetically trapped structures to be formed.^25,26^ Polymerization-induced self-assembly (PISA) emerged as an efficient way to prepare polymeric nanostructures by simultaneous polymerization and self-assembly, and it has also been conducted in flow for scale-up purposes.^27−30^ Benefiting from short optical path lengths in flow reactors, photo-PISA in flow was applied to access nanoparticles of various morphologies in the collaboration of Boyer, Zetterlund, and Junkers.^29,31^ By taking advantage of the high heat transfer rates in flow reactors, Warren and co-workers conducted all-aqueous PISA to realize rapid production of nanoparticles, where full conversion of monomer can be achieved in a short time and the morphologies can be tuned by the feed ratio.^28,30^ These studies highlight the suitability of flow chemistry for providing an efficient pathway to scale up living CDSA.

Herein, we present the first-ever transfer of living CDSA of PCL-based polymers from batch to flow reactors. The primary focus was to scale up production and investigate the influence of multiple mixing parameters on the morphology of nanoparticles. Continuous flow systems were developed, and the effect of temperature, flow rate, and residence time on the morphology of extended platelets was explored during epitaxial growth in flow (Tables S1–S5). Subsequently, the epitaxial growth of seeds in flow was performed, revealing distinct growth behaviors between seeds and platelets. Following this, a combination of two flow systems was utilized to prepare platelets with controllable sizes by easily adjusting the flow rates. Most importantly, platelets could be continuously collected under the predetermined mixing parameters, and the uniformity of flow platelets was significantly improved compared with the scaled-up batch counterparts.

Initially batch controls were established; living CDSA was conducted in 1 mL batch scale according to our previous report (Figures S1–S8).^11,12^ As expected, the incremental addition of the unimer corresponded to a proportional increase in the size of the platelets (Figure S8 and Table S7). After that, epitaxial growth of seeds was scaled up to 10 mL at a 1:10 seed/unimer ratio to investigate the reproducibility and scalability of batch living CDSA. The platelets prepared were similar in size to those made on a small scale (Figure S9 and Table S7, *A*_n_ = 0.65 μm^2^ for 10 mL and *A*_n_ = 0.69 μm^2^ for 1 mL). However, they exhibited a greater dispersity (*A*_w_/*A*_n_ = 1.27 for 10 mL and *A*_w_/*A*_n_ = 1.06 for 1 mL), which was attributed to the poor mixing conditions encountered at larger scales. At larger solution volumes, it takes longer to obtain a mixed homogeneous solution upon unimer addition. Due to the rapid rate of crystallization, these concentration gradients lead to platelets with a greater size dispersity. On the other hand, we attempted to scale up living CDSA by increasing the concentration of reactants to 10 times. However, the obtained platelets were of higher dispersity, and many extremely large platelets were observed (Figure S10). This was likely due to higher concentrations leading to a faster crystallization rate.^32−34^

Next, we transferred this living CDSA process to continuous flow platforms. The ultimate objective of this research was to develop a flow reactor system for the preparation of controllable platelets directly from seeds. However, it is important to acknowledge that living CDSA involves seeded growth followed by epitaxial growth; directly transferring both steps to the flow system without a comprehensive understanding of the underlying processes may result in uncontrollable outcomes. To simplify this process, the latter stage, the direct epitaxial growth of platelets, was initially conducted in our flow reactor to optimize conditions. These preprepared platelets are mentioned as original platelets to differentiate them from subsequent platelets after epitaxial growth in flow.

In the flow setup (Figure 1b), the original platelets (Figure 1a) and unimer solution were injected using syringe pumps, with the mass ratio of the original platelet/unimer controlled by their respective flow rates. To investigate the effect of temperature on the morphology of platelets, epitaxial growth of the original platelets was conducted at 19 °C (room temperature), 25 °C, and 30 °C. The total flow rate and the added amount of unimer was kept constant at 200 μL·min^–1^ and 25 equiv. Transmission electron microscopy (TEM) analysis of the platelets (Figure 1c–h, Table S8) clearly indicated that the size distribution of the platelets narrowed, indicating improved uniformity as temperature increased. There are two possible explanations for this observation: First, higher temperatures decreased the rate of crystallization,^35^ allowing for slightly longer mixing times, thereby increasing the uniformity of the extended platelets. Second, the laminar flow model exhibits a parabolic distribution of flow velocity, leading to significant layering of the fluid. Consequently, reactants between different layers experience limited diffusion, resulting in regional variations in the reactant concentration. However, higher temperatures promote the Brownian motion of molecules and enhance interlayer diffusion, leading to improved mixing and the generation of more uniform platelets. This speculation was further confirmed when repeating the experiment in a flow reactor with wider tubing (I.D. = 0.75 mm) at the same flow rate. Due to reduced laminar mixing in a wider tube, platelets with high size dispersity were observed even at 30 °C (Figure S12a). Therefore, to achieve highly uniform platelets, the following living CDSA work was performed at 30 °C in a narrow tube reactor (I.D. = 0.3 mm).

**Figure 1:**
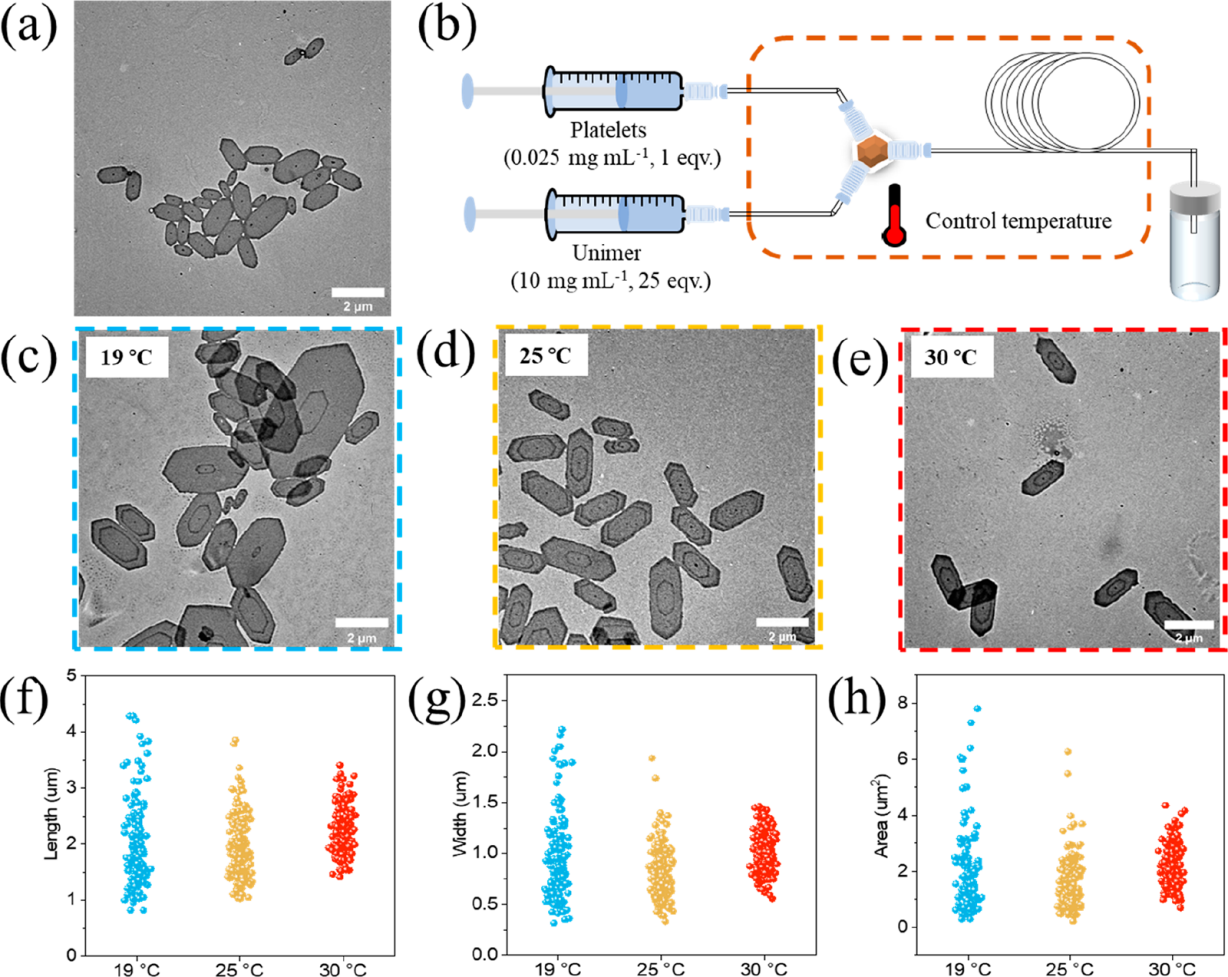
(a) TEM image of original platelets. (b) Illustration of flow setup for epitaxial growth of platelets. TEM images of platelets epitaxially grow at (c) 19 °C, (d) 25 °C, and (e) 30 °C. Platelet samples were not stained. Scale bars = 2 μm. Statistical value distribution of (f) length, (g) width, and (h) area.

Next the influence of the flow rate on the platelet size distribution was investigated. We expected that higher flow rates would lead to better mixing in the mixer as there is an increase in turbulence in the micromixer, and thus the dispersity would decrease.^36^ However, over the range of flow rates tested (100–800 μL·min^–1^), the shape and size of the extended platelets remained almost the same (Figure 2 and Table S9). This consistency suggests that adequate mixing was achieved across all of the flow rates. It can be substantiated by the rapid growth rate, with epitaxial growth completing within just 0.5 min at a flow rate of 800 μL·min^–1^. Inadequate mixing during this rapid growth phase could lead to concentration gradients, ultimately manifested as increased dispersity. However, it is worth considering that higher flow rates require higher pressure to counteract the resistance in the flow system, especially at elevated flow rates, which can increase the burden on the equipment. Therefore, for the subsequent experiments, the flow rate was maintained at 200 μL·min^–1^ to strike a balance between efficient production and manageable pressure requirements.

**Figure 2:**
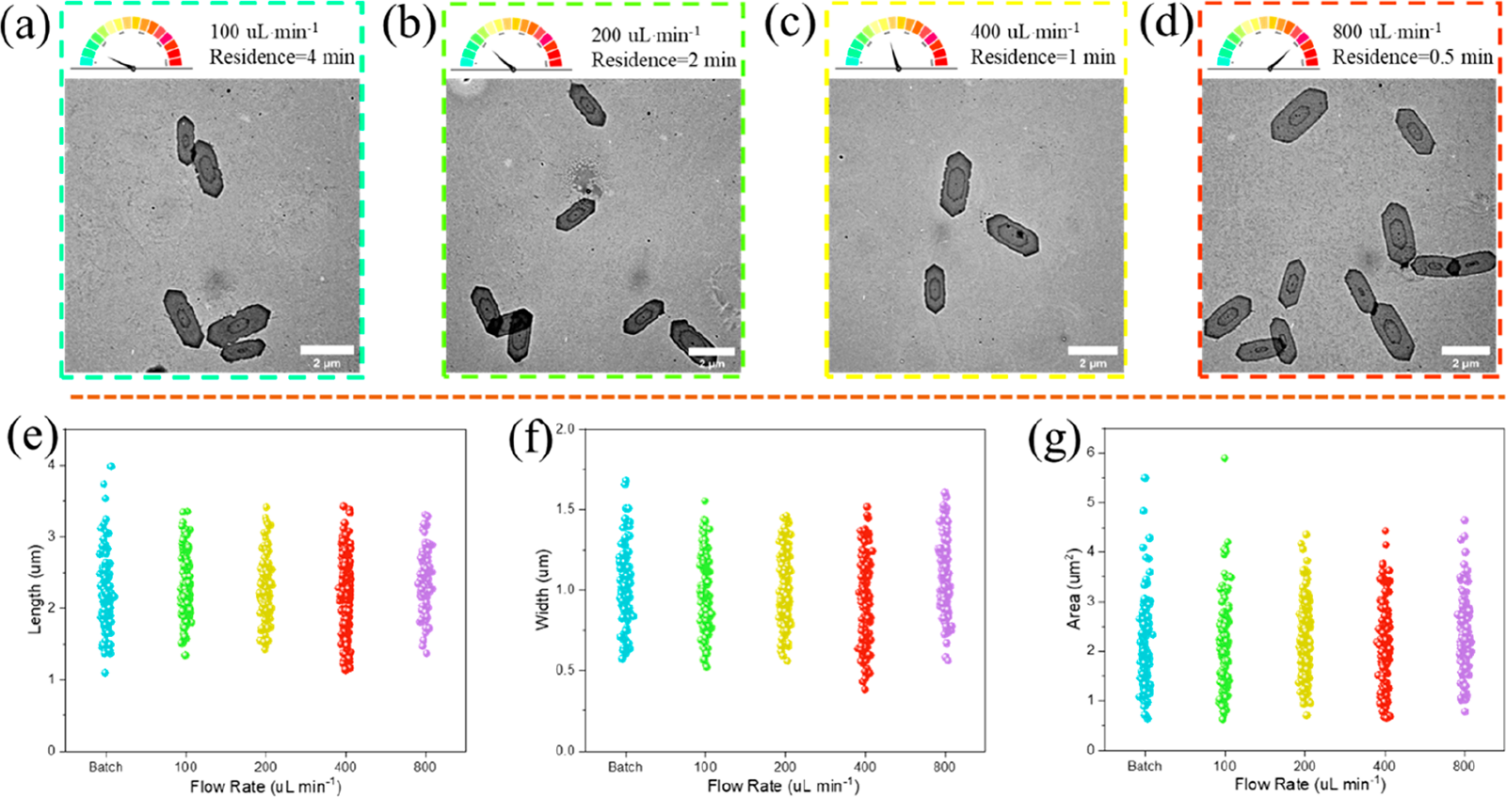
TEM images of platelets epitaxially grow at (a) 100 μL·min^–1^, (b) 200 μL·min^–1^, (c) 400 μL·min^–1^, and (d) 800 μL·min^–1^. Statistical value distributions of (e) length, (f) width, and (g) area.

After optimizing reactor conditions, the next step was to control the size of extended platelets. Different amounts of unimer ranging from 12.5 to 100 equiv of seeds were added by adjusting the flow rates of the original platelet and unimer solutions. Additionally, batch epitaxial growth experiments were conducted for comparison. As anticipated, the length, width, and area of the extended platelets in the flow reactor increased with increasing unimer equivalents (Figure 3, Figure S11, and Table S10). Notably, the area of the extended platelets exhibited a linear relationship with the unimer equivalents. These results were found to be comparable to those obtained from the batch reactor with a slightly narrower size dispersity seen for the flow system. This further validated the effectiveness of the continuous flow approach to control platelet size and dispersity compared to batch. To enhance clarity, all studied flow conditions and the size of corresponding platelets were summarized in Table S11.

**Figure 3:**
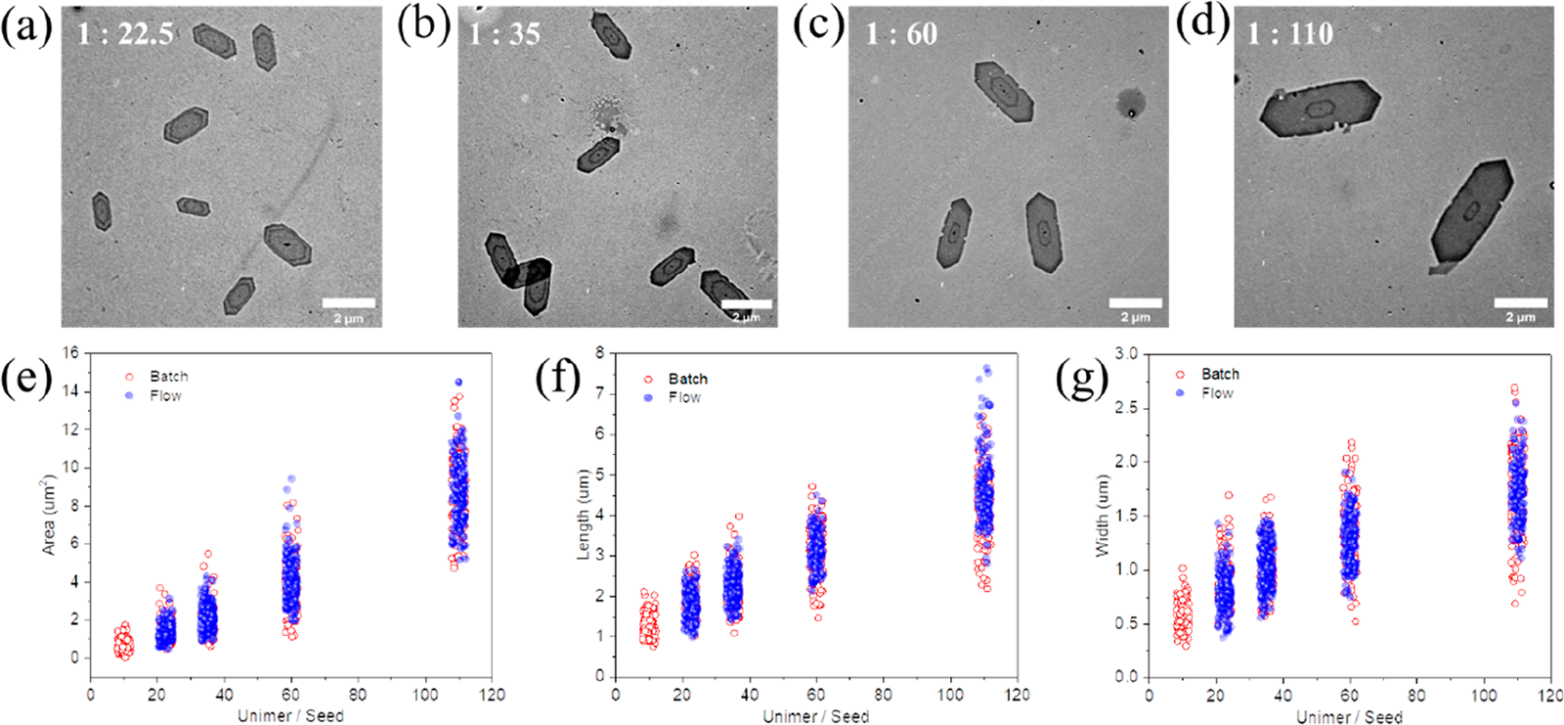
TEM images of flow extended platelets prepared at seed/unimer ratios of (a) 1:22.5, (b) 1:35, (c) 1:60, and (d) 1:110. Statistical comparison of platelets from flow and batch: (e) area, (f) length, and (g) width.

After optimizing the conditions, living CDSA was attempted using seed particles (Figure 4a). Initial attempts using seeds in the flow reactor differed significantly compared to the batch (Figure S12b). The presence of large, disperse platelets indicated the occurrence of self-nucleation of PCL homopolymer.^37,38^ These findings suggest there are different growth behaviors between seeds and platelets. Previous studies from our group demonstrated that the 1D seeds grow primarily from their ends,^12^ while 2D platelets can epitaxially grow in all directions, thus greater availability ensures unimer can be consumed quickly. Additionally, reduced corona density of the platelets, resulting from the addition of 50 wt % PCL homopolymer during the preparation of the original platelets, minimizes steric hindrance during crystallization, and thus epitaxial growth can be fast. Both reasons are conducive to reducing the occurrence of self-nucleation. If the unimer is not consumed quickly, then self-nucleation can occur, leading to rapid growth of undesired structures.

**Figure 4:**
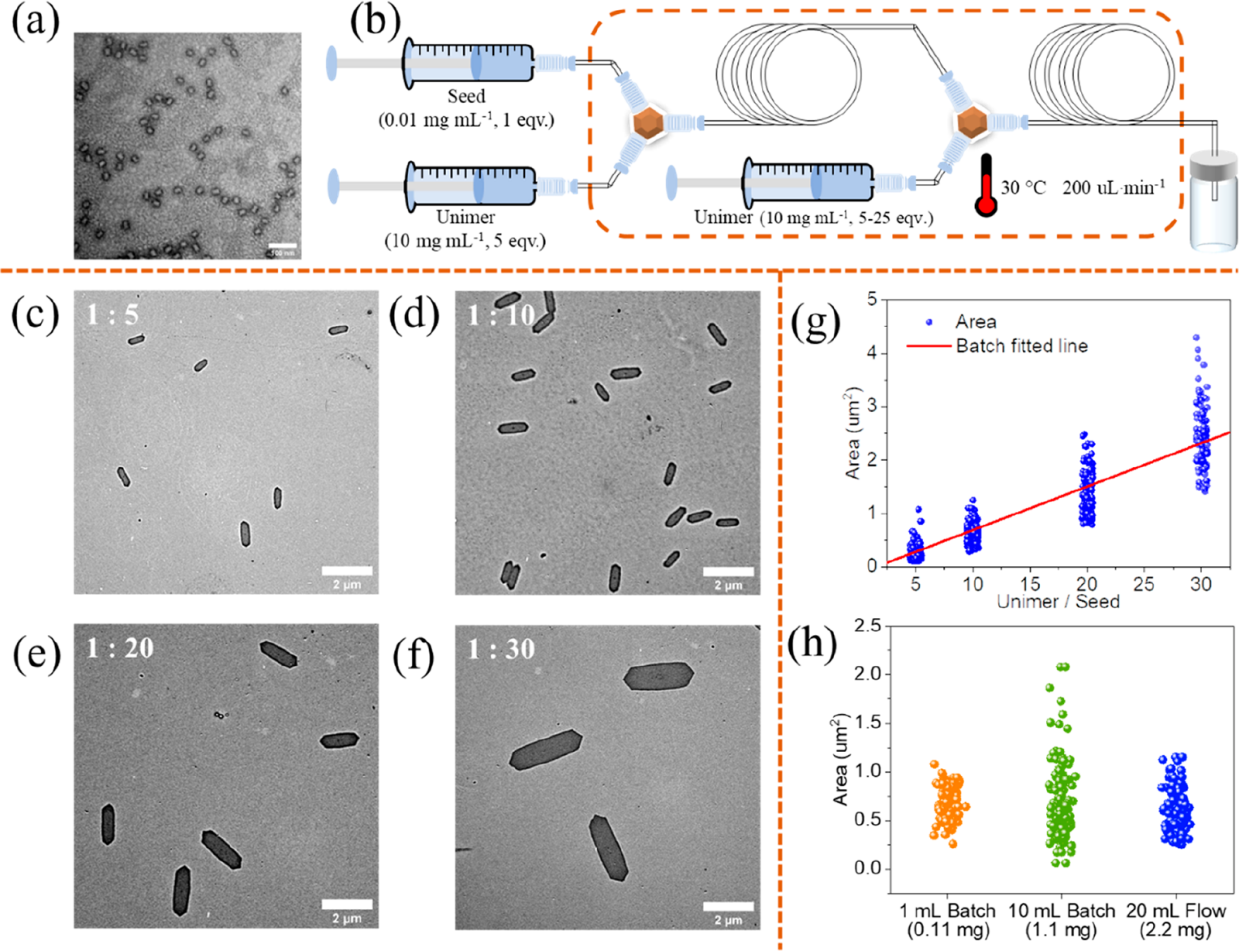
(a) TEM image of seeds. Sample was stained by uranyl acetate solution (1 wt %). (b) Illustration of flow setup for epitaxial growth of platelets. TEM images of flow platelets grown from seeds prepared at seed/unimer ratio of (c) 1:5, (d) 1:10, (e) 1:20, and (f) 1:30. (g) Area of flow platelets in comparison to the batch standards (red line). (h) Comparison of statistical size parameters of platelets prepared in different methods. Seed/unimer ratio is 1:10.

Given these potential causes of self-nucleation during the epitaxial growth of seeds, we proposed two possible solutions: (a) sonication of platelets to generate 2D seeds with a low corona density, which would consume unimer more efficiently, and (b) reducing the unimer/seed ratio to decrease the concentration of unimer, which was based on the observation that the rate of crystallization is highly influenced by the concentration.^32−34^ Even though large self-nucleation platelets were avoided when using platelet fragments as seeds, poor platelet uniformity (Figure S12d) was observed due to these 2D seeds (Figure S12c) being more disperse than those from 1D cylinders. However, when conducting living CDSA using seed particles at a lower unimer/seed ratio (5:1), where the rate of crystallization should be slower, no self-nucleated platelets were observed, and uniform platelets were obtained (Figure 4c).

After optimizing living CDSA from both seed and platelet particles, we then developed a flow reactor cascade (Figure 4b) capable of making multilayered platelets. Besides the epitaxial growth of seeds at the fixed unimer/seed ratio of 5:1, an additional living CDSA section was incorporated to control the size of the platelets. This combined approach involved the epitaxial growth of both seeds and platelets. Final platelet size was controlled by varying the amount of unimer added to the second living CDSA section, resulting in total unimer/seed ratios of 10:1, 20:1, and 30:1 (Figure 4d–f and Table S12). The size of the flow platelets increased as more unimer was added, and the area values showed a good fit to the batch controls (Figure 4g). For a more distinct presentation of the layered structure, fluorescence labeling was employed to highlight the second layer of platelets. As anticipated, this revealed hollow platelets (Figure S13), confirming the sequential execution of two distinct living CDSA processes in achieving size-controllable platelets. The advantages of flow reactors in terms of reproducibility and scalability were evident when comparing platelets prepared by using different methods. To illustrate it more intuitively, quality measurements were employed instead of volume. Although platelets prepared through different methods were of similar size, they differed significantly in uniformity (Figure 4h and Figure S14). When batch living CDSA was scaled up from 1 to 10 mL, although 1.1 mg platelets were obtained, the uniformity decreased considerably, with the standard deviation of area doubling (0.167 μm^2^ to 0.344 μm^2^), as well as for length and width. Comparatively, the uniformity of the 2.2 mg flow platelets, while slightly lower than the 0.11 mg platelets from the 1 mL batch scale, still surpassed that of the 10 mL batch scale despite being double the quantity. Furthermore, in terms of preparing platelets in large quantities, the flow reactor allowed for the continuous production of platelets of the same size and uniformity. In theory, even with higher output, the size and uniformity of flow platelets should remain consistent. This capability circumvented the sacrifice of uniformity that occurs in the scale-up of the batch counterpart. Attempts were made to further augment platelet yield by increasing concentration 10-fold. While 11 mg of platelets were successfully produced, the results closely resembled those obtained from the batch concentration increase, resulting in platelets with greater size dispersity (Figure S15). Nevertheless, the flow reactor demonstrated advantages in terms of reproducibility, scalability, and maintaining uniformity compared to the batch reactor for the preparation of platelets.

## Supporting Information Available

Materials, characterization techniques, experimental procedures, and additional data (NMR, SEC, TEM images) (PDF)
