# Supplementary material for: Enhancing the Scalability of Crystallization-Driven Self-Assembly Using Flow Reactors
Source: ACS Macro Lett. 2023 Nov 16;12(12):1636–41. doi: 10.1021/acsmacrolett.3c00600 (PMC10734305; doi:10.1021/acsmacrolett.3c00600)
Supplement: Supplementary file 1 — mz3c00600_si_001.pdf [file mz3c00600_si_001.pdf]

Supporting Information

Enhancing the scalability of crystallization-driven self-assembly using flow reactors

Laihui Xiao, Sam J. Parkinson, Tianlai Xia, Phillippa Edge, Rachel K. O'Reilly*

[a]School of Chemistry, University of Birmingham, Edgbaston, Birmingham B15 2TT, UK

*Corresponding Author: r.oreilly@bham.ac.uk (ROR)

Summary of Content:

Number of pages: 23

Number of tables: 11

Number of figures: 15

Number of schemes: 2

Materials

Sodium ethanethiolate (80%, Sigma Aldrich), carbon disulfide (≥99.9%, Sigma Aldrich), solid iodine (≥99.99%, Sigma Aldrich), 4,4'-azobis(4-cyanovaleric acid) (ACVA, ≥98%, Sigma Aldrich), borane tetrahydrofuran complex solution (1.0 M in THF, Sigma Aldrich), ethanol (anhydrous, ≥99.5%, Fisher Scientific), diethyl ether (anhydrous, ≥99.0%, Fisher Scientific), and chloroform ($CHCl_3$, anhydrous, ≥99%, Sigma Aldrich) were used as received. Dry tetrahydrofuran (THF, ≥99.9%, inhibitor-free, Sigma Aldrich) was purified through a solvent tower. ε-caprolactone (99%, ACROS Organics) was stored in the glovebox filled with N_2 after being dried with CaH_2 and then distilled. Diphenylphosphate (DPP, 99%, Sigma Aldrich) was dried using P_2O_5 before being kept in the glove box. 2,2'-azobis(2-methylpropionitrile) (AIBN, 98%, Sigma Aldrich) was recrystallized in methanol and stored at 4 °C in the dark before use. 1,4-dioxane (anhydrous, 99.8%, Fisher Scientific) and N, N-dimethyl acrylamide (DMA, 99%, Sigma Aldrich contains 500 ppm monomethyl ether hydroquinone as inhibitor) was purified with basic alumina every time before use.

Instrumentation

NMR spectra were recorded with a Bruker DPX-400 (400 MHz) spectrometer, and deuterated chloroform ($CDCl_3$) was used as the solvent. The obtained results were analysed and exported with MestReNova x64 software. SEC was conducted on Agilent 390-MDS equipment coupled with PLgel Mixed-D type columns, and the signals were detected by refractive index (RI) and ultraviolet (UV) detectors. Chloroform with 0.5% NEt_3 was used as eluent and the flow rate is 1 $mL \cdot min^{-1}$. Number-average (M_n) and weight-average (M_w) molecular weights and dispersity ($Đ_M$) were calibrated against polystyrene (PS) standards using Agilent SEC software. DSC was conducted using a Mettler Toledo HP DSC827 instrument. Samples of 5-10 mg in weight were placed in aluminium pans and heated and cooled between the temperature of -100 °C and 100 °C at 10 $°C \cdot min^{-1}$ under the N_2 atmosphere. Three cycles were carried out for each sample, and the data from the second cycle was analysed and collected. TEM images were

captured on a Jeol 1400 Bio TEM microscope with an acceleration voltage of 80 kV. Samples were prepared by drop cast 8 μL of solution on formvar-coated copper grids and then blotting away with filter paper. Another 8 μL of aqueous uranyl acetate solution (1 wt%) was dropped on the grid to stain the sample, and the excess was removed by filter paper. Samples were dried overnight in a desiccator box before testing. The obtained images were analysed by ImageJ, and at least 100 particles were analysed to calculate the average size. The number average length (L_n) and weight average length (L_w) were calculated according to equations (1) and (2), and the distribution was the value of L_w/L_n.

$$L_n = \frac{\sum_{i=1}^{n} N_i L_i}{\sum_{i=1}^{n} N_i} \quad (1) \qquad L_n = \frac{\sum_{i=1}^{n} N_i L_i^2}{\sum_{i=1}^{n} N_i L_i} \quad (2)$$

Similarly, the number average width (W_n), weight average width (W_w), number average area (A_n), and weight average area (A_w) were calculated according to equations (3) to (6), and their distributions were defined as W_w/W_n and A_w/A_n.

$$W_n = \frac{\sum_{i=1}^{n} N_i W_i}{\sum_{i=1}^{n} N_i} \quad (3) \qquad W_w = \frac{\sum_{i=1}^{n} N_i W_i^2}{\sum_{i=1}^{n} N_i W_i} \quad (4)$$

$$A_n = \frac{\sum_{i=1}^{n} N_i A_i}{\sum_{i=1}^{n} N_i} \quad (5) \qquad A_w = \frac{\sum_{i=1}^{n} N_i A_i^2}{\sum_{i=1}^{n} N_i A_i} \quad (6)$$

Flow reactors were set up by connecting several syringes with PTFE tubing (Figure 1a). Seeds and unimer (PCL / PCL-PDMA=1:1, 10 mg·mL^{-1} in $CHCl_3$) were stored in different syringes, and the weight ratios of seeds and unimer were tuned by their flow rates. To make sure seeds and unimer were well mixed, a Y-mixer was equipped at their intersection, where living CDSA started. Before entering the Y-mixer, seed solution was pre-heated to the setting temperature in a tubing coil (inner diameter: 0.8 mm, residence: 2 minutes), and then a cycle of living CDSA was completed once the mixture flowed through another tubing coil (inner diameter: 0.3 mm, residence: 2 minutes) after the Y-mixer. Both the mixer and the tubing coil were buried in the pre-heated sand bath.

To conduct more living CDSA sessions, more mixers and tubing coils could be connected in series, as illustrated in Figure 4b.

Synthesis

Synthesis of dual-headed chain transfer agent (CTA)

Synthesis of bis-(ethylsulfanylthiocarbonyl) disulfide

Carbon disulfide (7.74 mL) was added to a solution of sodium ethanethiolate (10 g) in diethyl ether (500 mL), which is immersed in an ice bath with a constant stir. After stirring for another 2 hours, excess iodine was added to the flask until the colour of the reaction system became dark brown. The solution was kept stirred overnight, and then it was washed with a solution of sodium thiosulfate (1M, 3 × 100 mL) followed by brine (3 × 100 mL) after it was transferred into a separation funnel. The solvent was removed by rotary evaporation after the organic layer was dried with anhydrous magnesium sulfate. 15.27 g (yield: 93.7%) of an orange liquid was obtained.

^{1}H NMR (400 MHz, Chloroform-d, δ (ppm)): 3.32 (q, J = 7.5 Hz, 2H, $SC\boldsymbol{H}_2CH_3$), 1.36 (t, J = 7.5 Hz, 3H, $SCH_2C\boldsymbol{H}_3$).

Synthesis of 4-cyano-4-[(ethylsulfanylthiocarbonyl)sulfanyl] pentanoic acid (CEPA)

Bis-(ethylsulfanylthiocarbonyl) disulfide (9.0265 g, 32.88 mmol), ACVA (13.8245 g, 49.32 mmol), and ethyl acetate (500 mL) were charged into a 1-L round bottle flask equipped with a condenser, and the reaction was heated to reflux overnight in an N_2 atmosphere. After being concentrated by rotary evaporation, the crude product was purified through silica gel column chromatography (EtOAc / DCM = 1:3). Finally, 21.58 g (yield: 73.7%) of an orange solid was obtained.

^{1}H NMR (400 MHz, Chloroform-d, δ (ppm)): 3.35 (q, J = 7.4 Hz, 2H, $SC\boldsymbol{H}_2CH_3$), 2.73 – 2.62 (m, 2H, $C\boldsymbol{H}_2COOH$), 2.62 – 2.48 (m, 1H, $C\boldsymbol{H}_2CH_2COOH$), 2.40 (m, 1H, $C\boldsymbol{H}_2CH_2COOH$), 1.89 (s, 3H, $C\boldsymbol{H}_3C$), 1.36 (t, J = 7.4 Hz, 3H, $SCH_2C\boldsymbol{H}_3$).

Synthesis of 2-cyano-5-hydroxypentan-2-yl-ethyl carbonotrithioate (CHPET)

CEPA (3 g, 11.4 mmol) was charged into a two-neck flask coupled with a dropping flask, and then the reaction apparatus was kept in the nitrogen flow and cooled to -78 °C with a dry ice – acetone bath. Dry tetrahydrofuran was used as the solvent and added to the flask with a syringe, and borane tetrahydrofuran (12 mL, 0.12 mmol) was dropped into the solution subsequently. After an hour of reaction at -78 °C, the solvent was left at room temperature and kept stirred overnight. 2-propanol was added to react with the extra borane. After the reaction, the solvent was removed by rotary evaporation, and diethyl ether (200 mL) was added to dissolve the crude product. The solution was washed with saturated $NaHCO_3$ (3 × 200 mL) and then with brine (1 × 200 mL). The organic layer was concentrated by rotary evaporation after it was dried by anhydrous $MgSO_4$. Silica gel column chromatography (n-hexane / EtOAc = 1:1) was used to purify the product further, and 2.13 g (yield: 74.9%) of orange oil was finally obtained. The synthesis route to access CTA was illustrated in Scheme S1.

^{1}H NMR (400 MHz, Chloroform-d, δ (ppm)): 3.72 (t, J = 6.1 Hz, 1H, C***H***$_2$OH), 3.35 (q, J = 7.4 Hz, 2H, SC***H***$_2$CH$_3$), 2.34 – 2.22 (m, 1H, C(CN)(CH$_3$)C***H***$_2$CH$_2$), 2.16 – 2.06 (m, 1H, C(CN)(CH$_3$)C***H***$_2$CH$_2$), 1.90 (s, 3H, C(CN)(C***H***$_3$)CH$_2$CH$_2$), 1.89 – 1.79 (m, 2H, C(CN)(CH$_3$)CH$_2$C***H***$_2$), 1.36 (t, J = 7.4 Hz, 3H, SCH$_2$C***H***$_3$).

^{13}C NMR (101 MHz, Chloroform-d, δ (ppm)): 217.39 (***C***=S), 119.57 (C(***C***N)(CH$_3$)CH$_2$CH$_2$), 61.80 (***C***H$_2$OH), 46.99 (***C***(CN)(CH$_3$)CH$_2$CH$_2$), 35.76 (C(CN)(CH$_3$)***C***H$_2$CH$_2$), 31.31 (S***C***H$_2$CH$_3$), 27.91 (C(CN)(CH$_3$)CH2***C***H$_2$), 24.93 (C(CN)(***C***H$_3$)CH$_2$CH$_2$), 12.80 (SCH$_2$***C***H$_3$).

Synthesis of polymers

Polycaprolactone (PCL_{50}) synthesized by ring-opening polymerization

Ring-opening polymerization was conducted in a glove box filled with N_2. Briefly, caprolactone (2.246g, 19.68 mmol), diphenylphosphate (DPP, 70.33 mg, 0.28 mmol), and CHPET (70 mg, 0.28 mmol) were weighed accurately in vials and then transferred to a 50-mL round bottle flask. Toluene (19.726 mL) was used as the solvent for the reaction. Polymerization was conducted at room temperature, and ^{1}H NMR was used to monitor the process. After about 6.5 h, the reaction was quenched by the Amberlyst

agent, and the solution was removed from the glove box. The crude product was precipitated into cold diethyl ether 3 times and collected after centrifugation. 1.34 g (monomer conversion: 71%) of light-yellow solid was obtained before it was dry in a vacuum oven.

^{1}H NMR (400 MHz, Chloroform-d, δ (ppm)): 4.06 (t, J = 6.7 Hz, 2H, COOC$\boldsymbol{H}_2$), 2.30 (t, J = 7.5 Hz, 2H, C$\boldsymbol{H}_2$COO), 1.65 (dtt, J = 13.4, 6.7, 3.7 Hz, 4H, COOCH$_2$C$\boldsymbol{H}_2$CH$_2$C$\boldsymbol{H}_2$CH$_2$OH), 1.44 – 1.32 (m, 2H, (CH2)$_2$C$\boldsymbol{H}_2$(CH$_2$)$_2$).

Polycaprolactone-b-polydimethylacetamide (PCL$_{50}$-PDMA$_{196}$) synthesized by RAFT polymerization

Macro-CTA (PCL$_{50}$, 100 mg, 0.0168 mmol), DMA (399.7 mg, 4.032 mmol), and AIBN (0.276 mg, 0.00168 mmol, 10 mg mL^{-1} in dioxane) were dissolved in dioxane (1 mL) and then transferred into an ampoule. The solution was freeze-pump-thawed three times before the ampoule was immersed in an oil bath set at 70 °C. After 2 hours, polymerization was quenched by immersing the ampoule in the liquid N$_2$. The crude product was precipitated into the cold diethyl ether once it reached room temperature and then collected by centrifugation, and this process was repeated three times. After drying in a vacuum oven for 3 days, 458.5 mg (monomer conversion: 80%) of a solid product was obtained.

^{1}H NMR (400 MHz, Chloroform-d, δ (ppm)): 4.06 (t, J = 6.7 Hz, 2H, COOC$\boldsymbol{H}_2$), 3.21 – 2.75 (m, 6H, CON(C$\boldsymbol{H}_3$)$_2$), 2.30 (t, J = 7.5 Hz, 1H, C$\boldsymbol{H}_2$COO), 1.71 – 1.58 (m, 4H, COOCH$_2$C$\boldsymbol{H}_2$CH$_2$C$\boldsymbol{H}_2$CH$_2$OH), 1.44 – 1.32 (m, 2H, (CH2)$_2$C$\boldsymbol{H}_2$(CH$_2$)$_2$).

PCL modified by bodipy 630/650

Bodipy 630/650 was grafted to PCL50 by esterification reaction, which was catalyzed by N,N′-dicyclohexylcarbodiimide (DCC) and 4-(dimethylamino)pyridine (DMAP). In brief, PCL$_{50}$ (100 mg, 0.0168 mmol), bodipy 630/650 (3.78 mg, 0.0084 mmol), DCC (34.64 mg, 0.168 mmol), DMAP (2.05 mg, 0.0168 mmol), and DCM (2 mL) were added into a vial and stirred at room temperature for 2 days. After the reaction, the undissolved substance was removed by filtration, and the filtrate was precipitated into

the cold diethyl ether and then the precipitated PCL polymer was collected after centrifugation. Two other precipitation and centrifugation processes were conducted, and the final product was dried in a vacuum oven overnight.

General procedure for CDSA

Cylinders prepared by direct crystallisation-driven self-assembly (CDSA)

PCL_{50}-b-$PDMA_{196}$ (10 mg) and ethanol (2 mL) were measured accurately and added into a 7-mL vial. The mixture was heated at 70 °C for 3 hours without stirring and then left to cool down naturally. After ageing for 7 days at room temperature, a cloudy cylinder solution was obtained.

Seeds prepared by sonicating cylinders

The original solution of cylinders was diluted to 0.5 mg·mL^{-1}, and then 3 mL of the diluted solution was transferred into a quartz tube and immersed in the dry ice-acetone bath during the whole sonication process. A total of 20 min sonication was applied by a sonic probe with the model of 60 cycles of a 20-second sonication followed by a 100-second pause. The obtained solution of seeds was transferred to a vial for storage and imaged through TEM to determine the average size.

General procedure of living CDSA to prepare platelets in batch

The solution of seeds was diluted to 0.01, and then in the 1-mL scale, the unimer solution (10 mg·mL^{-1} of the 1:1 mixture in weight of homo and diblock polymers in $CHCl_3$) of a pre-calculated amount was added. After a 5-second handshake followed by 2-minute ageing at room temperature, samples were prepared for TEM or AFM, and the average size of platelets was measured according to the obtained images. For the batch scale-up, a 10-mL scale living CDSA was conducted using the same procedure.

General procedure of living CDSA to prepare platelets in the flow reactor

Seeds (or original platelets) and unimer were loaded into syringes, and then the mixer and coil were buried in the sand bath at a pre-set temperature and equilibrated for 30 minutes before the flow started. The flow rates of seeds and unimer were set up according to Table 1-5. Every time flow rates, seeds, or unimer were changed, samples were collected after passing 3 reactor volumes to make sure they reached the steady state. TEM samples were prepared instantly once platelets were collected. The detailed flow setups for different experiments were listed in Table S1-5.

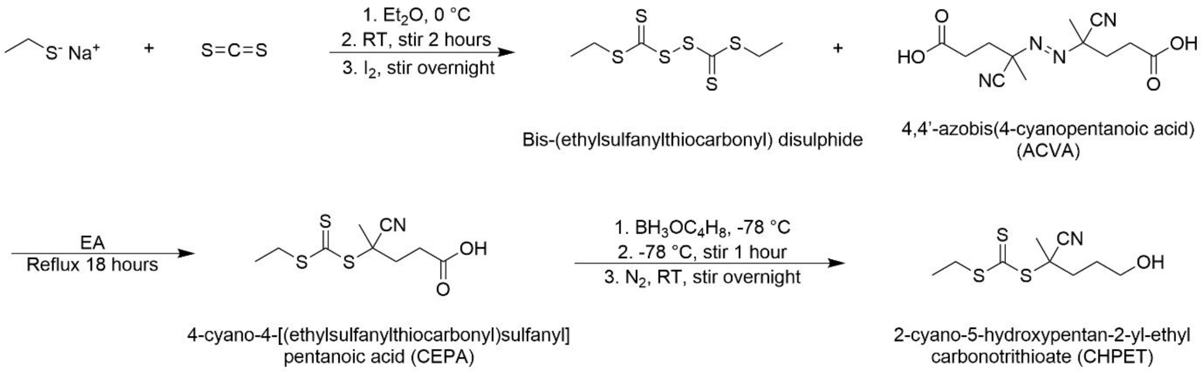

Scheme S1. The synthetic route of CHPET.

Table S1. Setup of flow reactor for epitaxial growth of platelets at various temperatures.

Sample	Temperature (°C)	Unimer (eqv. of seed)	R_p [a] (μL·min^{-1})	R_u [a] (μL·min^{-1})	Time [b] (min)
1	19	25	198	2	2
2	25	25	198	2	2
3	30	25	198	2	2

[a] R_p and R_u are abbreviations of the flow rate of original platelets and unimer, and [b] T is the time solution flows through the coil. The concentrations of original and unimer are fixed at 0.04 and 10 mg·mL^{-1} respectively in this work.

Table S2. Setup of flow reactor for epitaxial growth of platelets at various flow rates.

Sample	R_p (μL·min^{-1})	R_u (μL·min^{-1})	Unimer (eqv. of seed)	Temperature (°C)	Time (min)	*Re**
1	99	1	25	30	4	5.8
2	198	2	25	30	2	11.5
3	396	4	25	30	1	23.0
4	792	8	25	30	0.5	46.0

*Re is the abbreviation of Reynolds number, which is calculated by $Re = \rho v d/\mu$. ρ, v, d, and μ are density, flow rate, diameter of tubing, and viscosity respectively.

Table S3. Setup of flow reactor for epitaxial growth of platelets with various amounts of unimer.

Sample	Unimer (eqv. of seed)	R_p (μL·min^{-1})	R_u (μL·min^{-1})	Temperature (°C)	T (min)
1	12.5	198	2	30	2
2	25	198	2	30	2
3	50	198	2	30	2
4	100	198	2	30	2

Table S4. Setup of flow reactor for epitaxial growth of seeds with various amounts of unimer.

Sample	Unimer (eqv. of seed)	R_s [a] (μL·min^{-1})	R_u (μL·min^{-1})	Temperature (°C)	Time (min)
1	10	198	2	30	2
2	5	199	1	30	2
3*	10	198	2	30	2

[a] R_s is the abbreviation of the flow rate of seeds, and " * " indicates the seeds were prepared by sonicating platelets.

Table S5. Setup of flow reactor to prepare size-controllable platelets from seeds

Sample	Total unimer (eqv. of seed)	1st section Unimer (eqv. of seed)	1st section R_s (μL·min^{-1})	1st section R_u (μL·min^{-1})	2nd section Unimer (eqv. of seed)	2nd section R_u (μL·min^{-1})	Time (min)
1	5	5	199	1			2
2	10	5	199	1	5	1	4
3	20	5	199	1	15	3	4
4	30	5	199	1	25	5	4

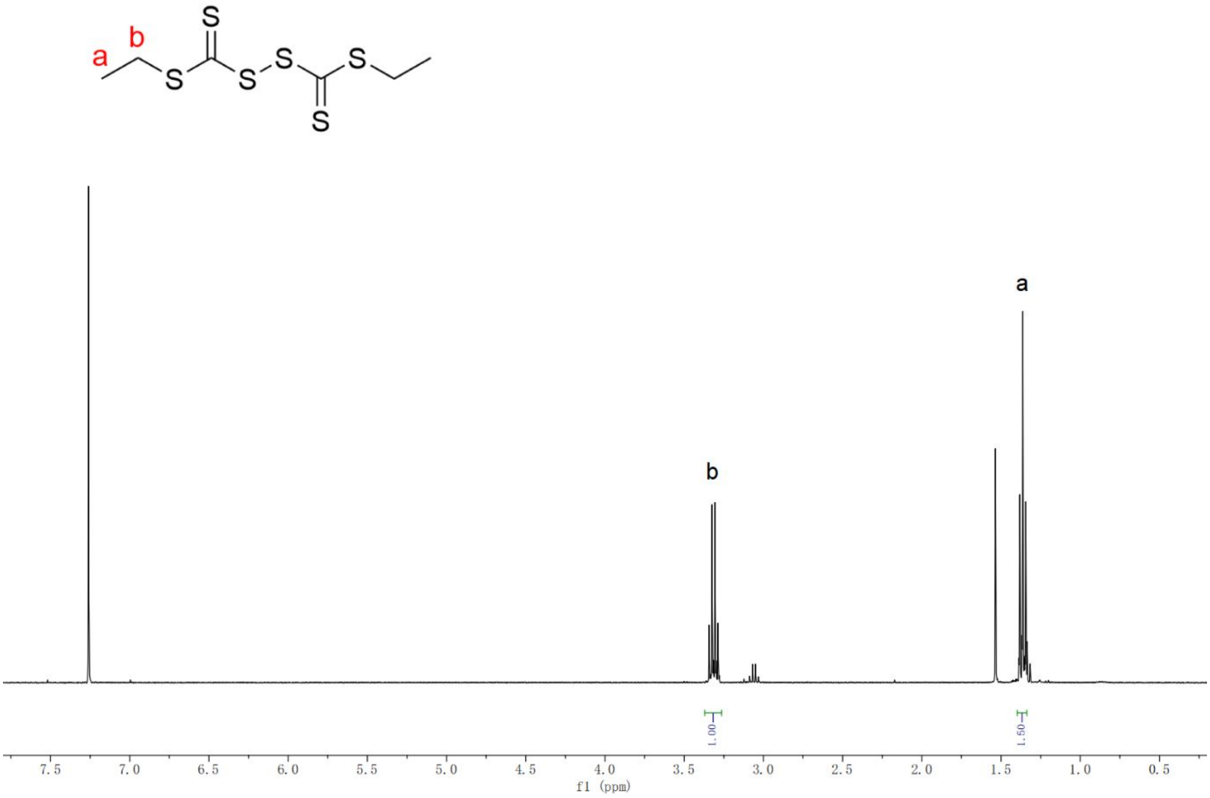

Figure S1. ^{1}H NMR spectrum of bis-(ethylsulfanylthiocarbonyl) disulfide (400 MHz, in $CDCl_3$).

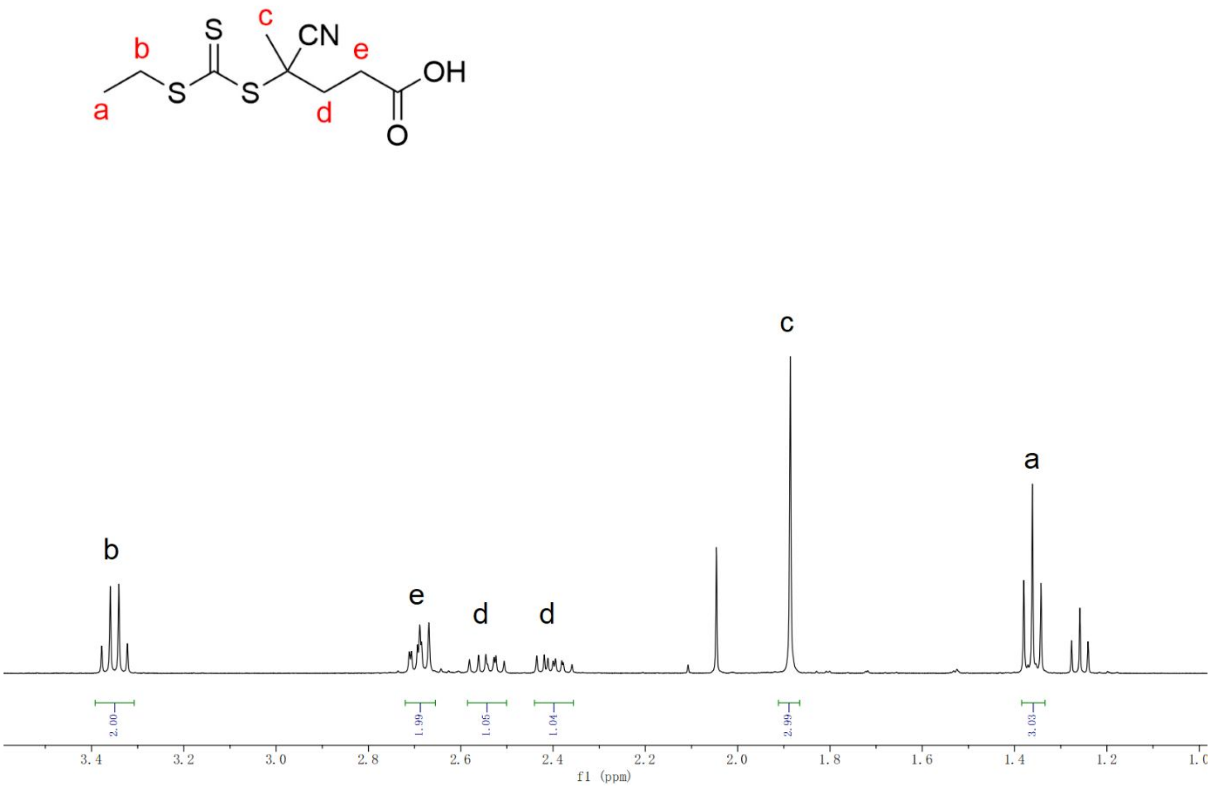

Figure S2. ^{1}H NMR spectrum of CEPA (400 MHz, in $CDCl_3$).

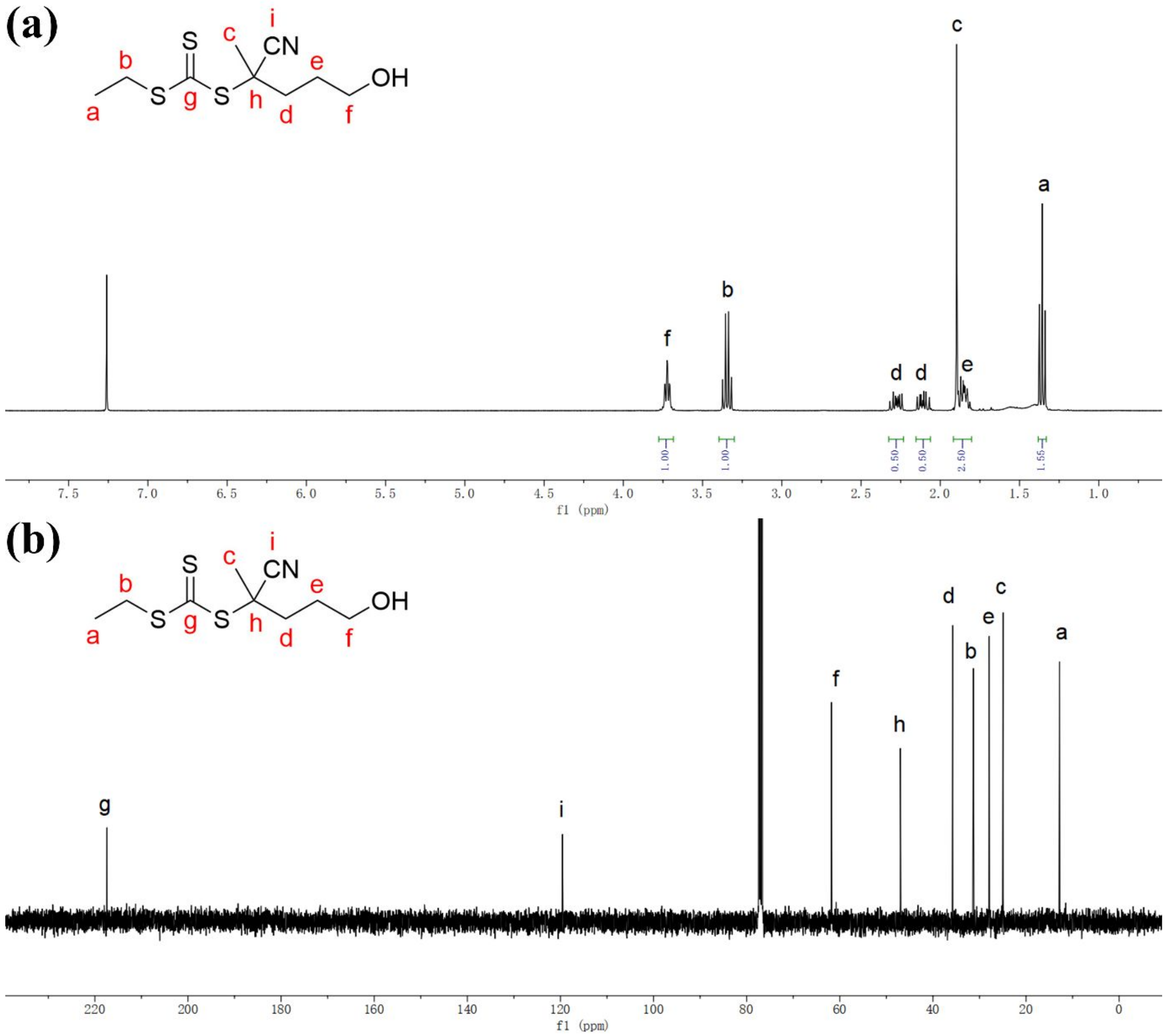

Figure S3. ^{1}H (a) and ^{13}C (b) NMR spectrum of CEPET (400 MHz, in $CDCl_3$).

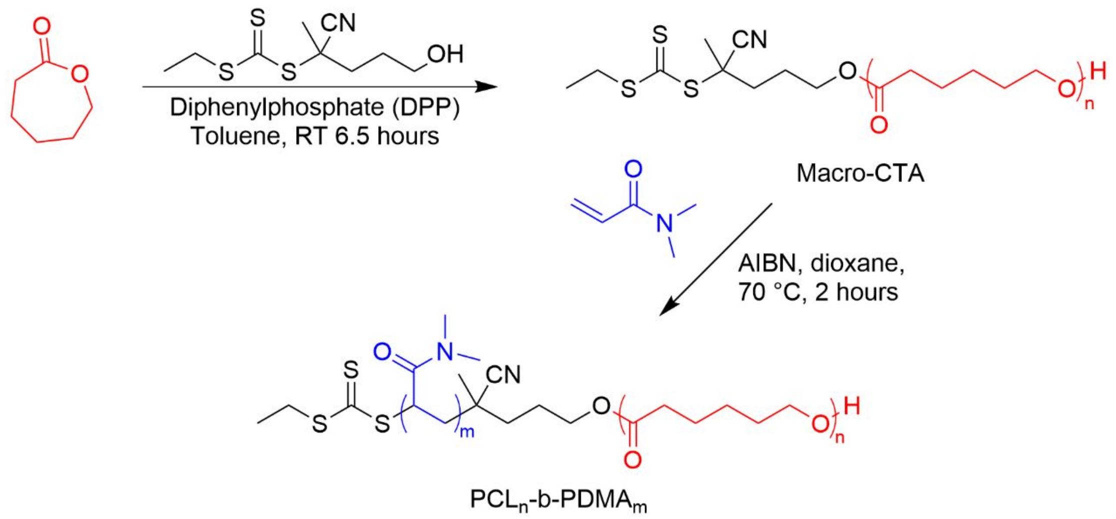

Scheme S2. Synthetic route of PCL-b-PDMA.

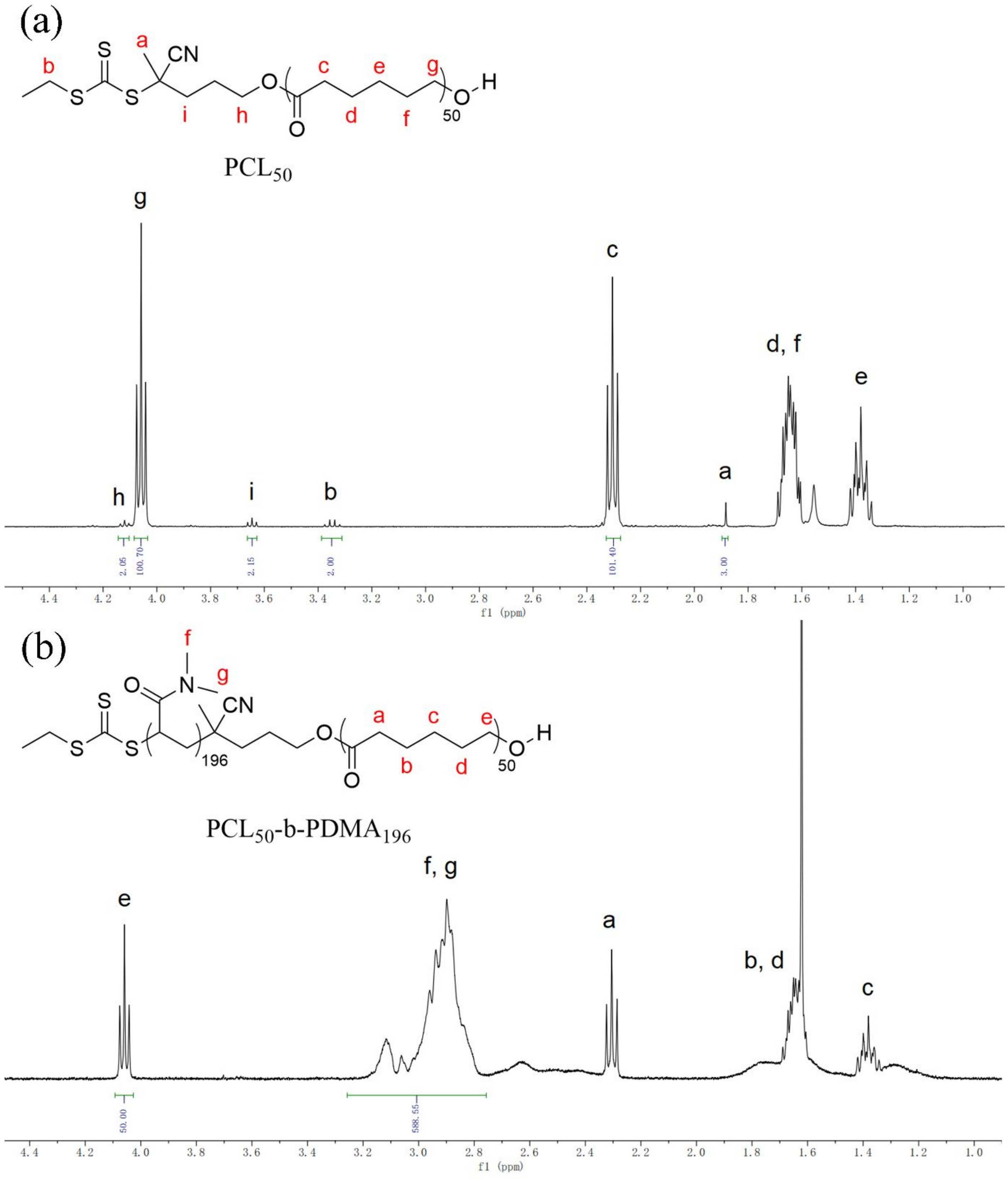

Figure S4. ^{1}H NMR spectra of (a) PCL_{50} and (b) PCL_{50}-b-$PDMA_{196}$ (400 MHz, in $CDCl_3$).

Table S6. SEC results of polymers detected by RI.

Polymer	M_n (kg·mol^{-1})	M_w (kg·mol^{-1})	$Đ$
PCL_{50}	13.3	14.2	1.06
PCL_{50}-b-$PDMA_{196}$	33.0	37.5	1.14

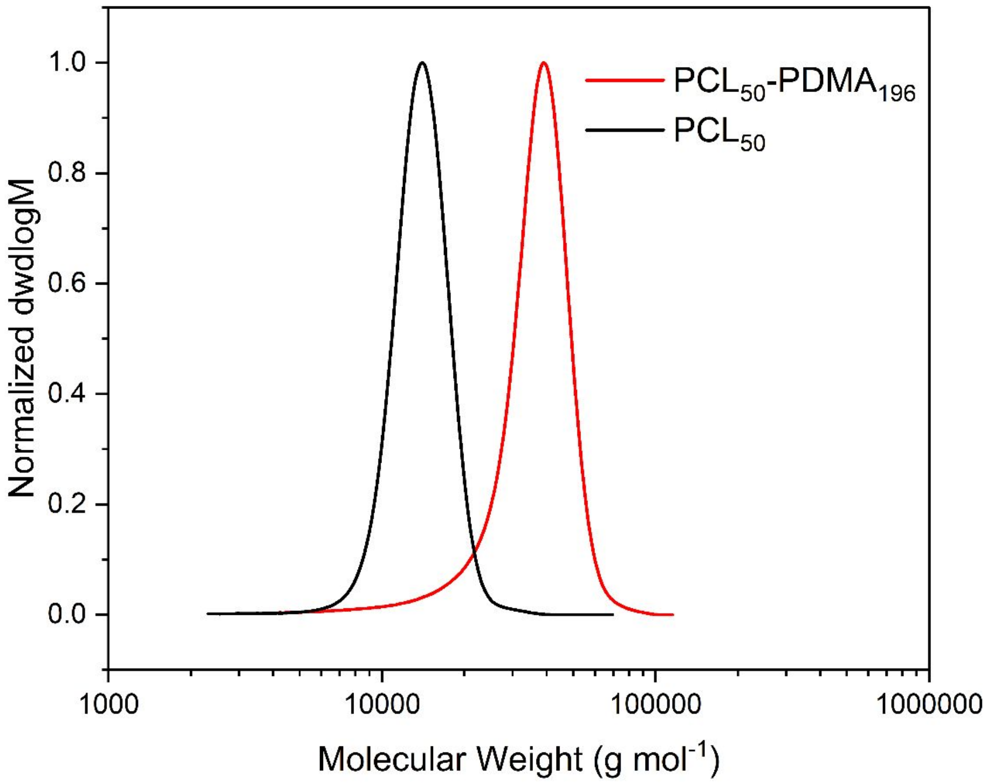

Figure S5. Normalized SEC curves of PCL_{50} and PCL_{50}-b-$PDMA_{196}$.

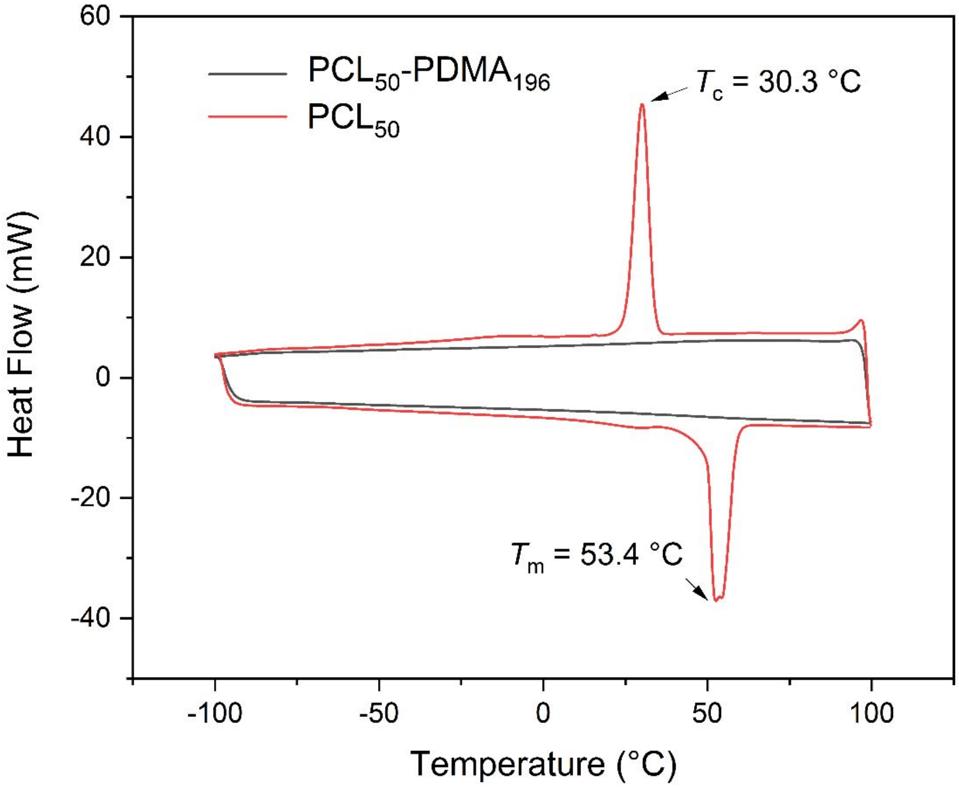

Figure S6. DSC thermogram of PCL_{50} and PCL_{50}-$PDMA_{196}$.

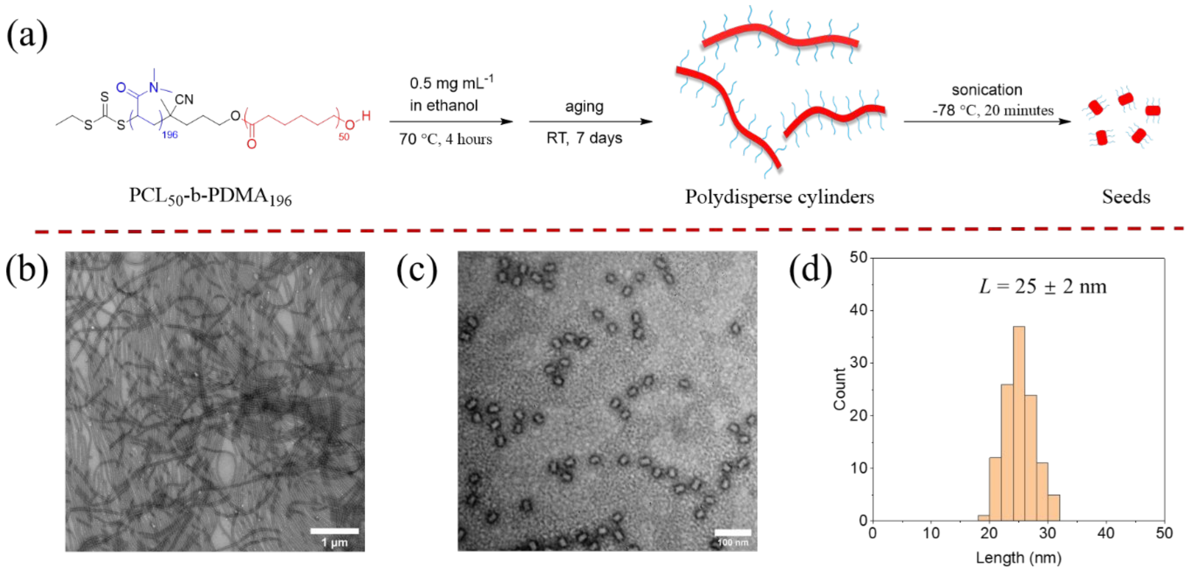

Figure S7. (a) Scheme to prepare seeds from PCL-PDMA. TEM images of (b) polydisperse cylinders and (c) seeds. Uranyl acetate aqueous solution (1%) was used for stain. (d) Length distribution of seeds. At least 100 particles were analysed to obtain statistical results.

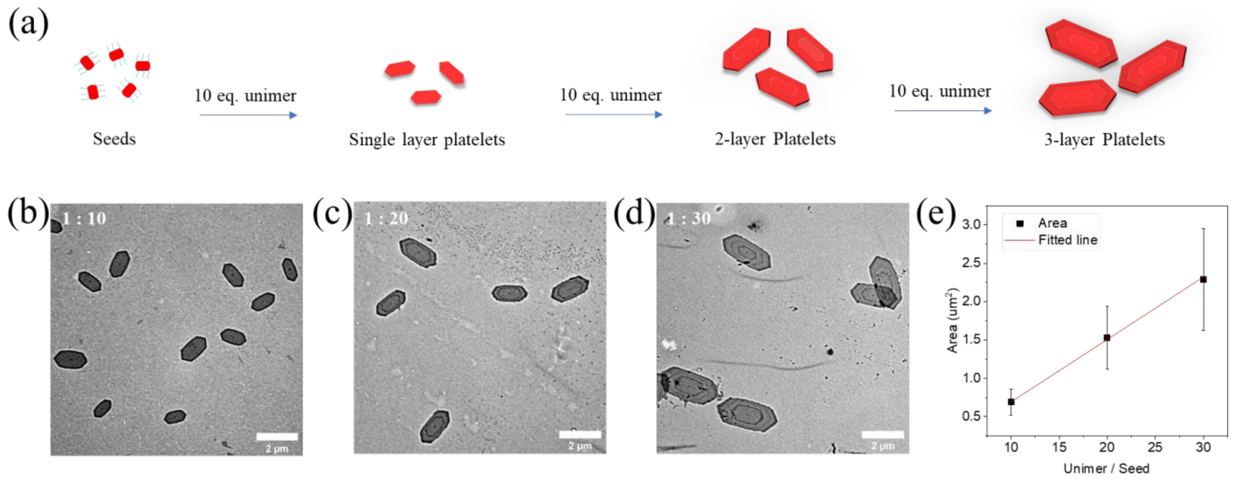

Figure S8. (a) Scheme of living CDSA. TEM images of platelets prepared at seed/unimer ratio of (b) 1:10, (c) 1:20, and (d) 1:30. (e) Linear fit of area upon different unimer/seed ratios. Error bars represent the standard deviation of the area distribution.

Table S7. Batch platelets prepared using various amount of unimer.

Unimer	Area (um^2)			Length (um)			Width (um)			
(eqv. of seed)	A_n	A_w	A_w/A_n	L_n	L_w	L_w/L_n	W_n	W_w	W_w/W_n	L/W
10	0.69	0.73	1.06	1.25	1.28	1.02	0.60	0.62	1.03	2.09
20	1.53	1.64	1.07	1.96	1.99	1.02	0.87	0.89	1.02	2.24
30	2.29	2.48	1.08	2.32	2.37	1.02	1.01	1.04	1.03	2.30
10*	0.65	0.83	1.27	1.24	1.31	1.04	0.58	0.62	1.05	2.14

*Platelets were prepared at 10 mL scale.

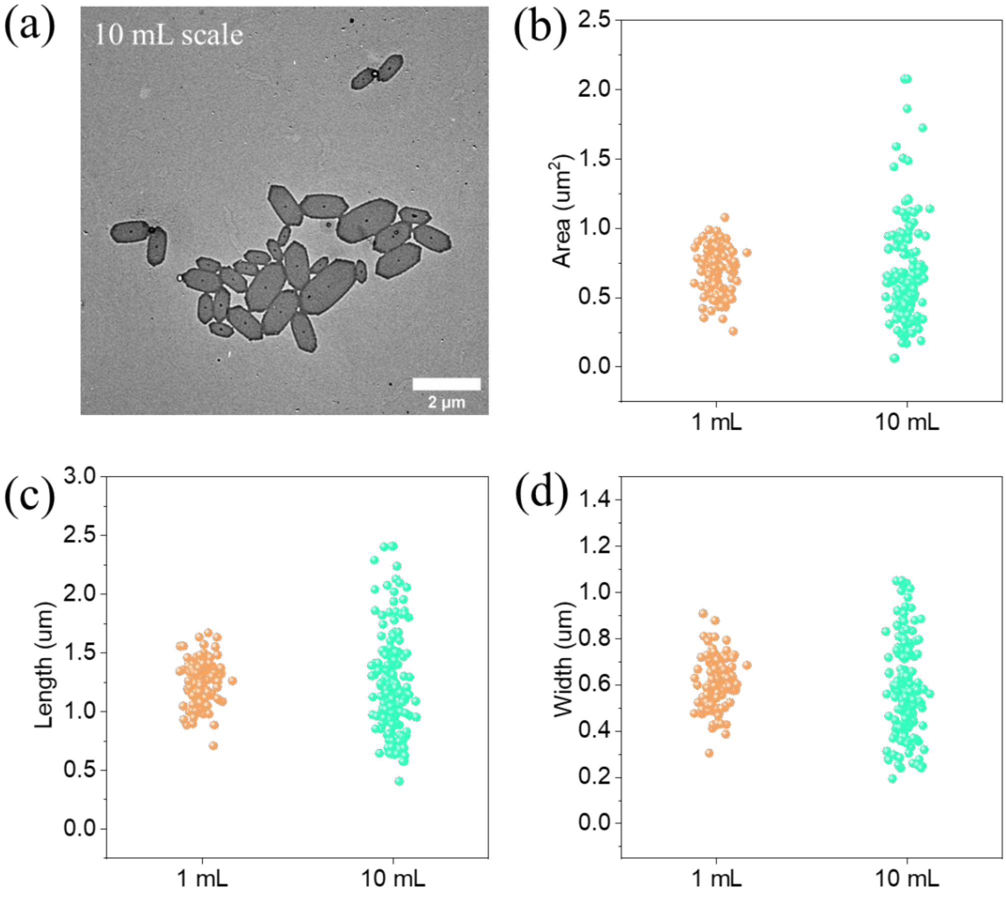

Figure S9. Size comparison of platelets from 1-mL and 10-mL scales: (a) TEM image of platelets prepared from the 10 mL scale, (b) area, (c) length, and (d) width.

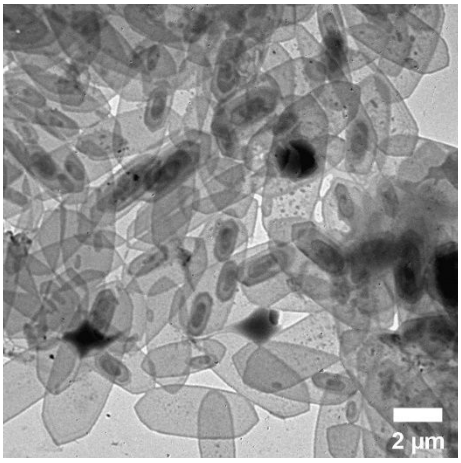

Figure S10. TEM image of platelets prepared at 1 mg mL^{-1} in 1 mL batch scale.

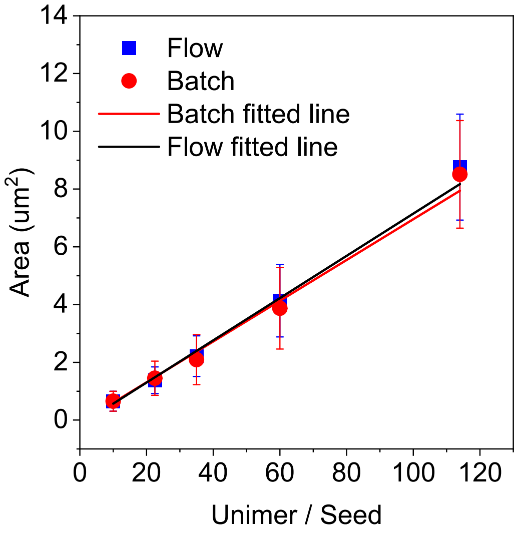

Figure S11. Linear fitting of area and unimer/seed ratio. The error bar represents the standard deviation.

Table S8. Extended flow platelets prepared using original platelets as seeds at various temperature.

Temperature	Area (um^2)			Length (um)			Width (um)			
(°C)	A_n	A_w	A_w/A_n	L_n	L_w	L_w/L_n	W_n	W_w	W_w/W_n	L/W
19	1.88	2.97	1.58	2.00	2.27	1.13	0.97	1.14	1.17	2.06
25	1.51	2.01	1.33	1.91	2.06	1.08	0.82	0.89	1.09	2.33
30	2.21	2.44	1.10	2.27	2.33	1.03	1.02	1.06	1.04	2.23

Table S9. Extended flow platelets prepared using original platelets as seeds at various flow rates.

Flow rate (μL·min^{-1})	Time spent (min)	Area (um^2)			Length (um)			Width (um)			
		A_n	A_w	A_w/A_n	L_n	L_w	L_w/L_n	W_n	W_w	W_w/W_n	L/W
100	4	2.05	2.39	1.16	2.26	2.34	1.04	0.98	1.03	1.05	2.30
200	2	2.21	2.44	1.10	2.27	2.33	1.03	1.02	1.06	1.04	2.23
400	1	2.11	2.44	1.15	2.24	2.37	1.06	0.96	1.04	1.08	2.33
800	0.5	2.33	2.56	1.10	2.40	2.47	1.03	1.10	1.14	1.04	2.19

Table S10. Extended platelets prepared using original platelets as seeds with various amount of unimer both in flow and batch reactors.

	Unimer	Area (um^2)			Length (um)			Width (um)			
	(eqv. of seed)	A_n	A_w	A_w/A_n	L_n	L_w	L_w/L_n	W_n	W_w	W_w/W_n	L/W
Flow	12.5	1.38	1.53	1.11	1.77	1.85	1.04	0.80	0.85	1.06	2.21
	25	2.21	2.44	1.10	2.27	2.33	1.03	1.02	1.06	1.04	2.23
	50	4.13	4.51	1.09	3.13	3.21	1.03	1.27	1.32	1.03	2.46
	100	8.76	9.14	1.04	4.78	4.99	1.04	1.73	1.78	1.03	2.76
Batch	12.5	1.45	1.69	1.17	1.89	1.99	1.05	0.93	0.98	1.06	2.05
	25	2.09	2.45	1.17	2.19	2.30	1.05	1.02	1.07	1.05	2.16
	50	3.87	4.38	1.13	3.08	3.21	1.04	1.38	1.45	1.05	2.23
	100	8.51	8.91	1.05	4.31	4.48	1.04	1.70	1.79	1.05	2.54

Table S11. Summary table of all condition studied for flow living CDSA and the size of the corresponding platelets.

	R_t (μL·min^{-1})	Time (min)	Temperature (°C)	Unimer (eqv. of seed)	Area (um^2)			Length (um)			Width (um)			
					A_n	A_w	A_w/A_n	L_n	L_w	L_w/L_n	W_n	W_w	W_w/W_n	L/W
Flow	200	2	19	25	1.88	2.97	1.58	2.00	2.27	1.13	0.97	1.14	1.17	2.06
	200	2	25	25	1.51	2.01	1.33	1.91	2.06	1.08	0.82	0.89	1.09	2.33
	200	2	30	25	2.21	2.44	1.10	2.27	2.33	1.03	1.02	1.06	1.04	2.23
	100	4	30	25	2.05	2.39	1.16	2.26	2.34	1.04	0.98	1.03	1.05	2.30
	400	1	30	25	2.11	2.44	1.15	2.24	2.37	1.06	0.96	1.04	1.08	2.33
	800	0.5	30	25	2.33	2.56	1.10	2.40	2.47	1.03	1.10	1.14	1.04	2.19
	200	2	30	12.5	1.38	1.53	1.11	1.77	1.85	1.04	0.80	0.85	1.06	2.21
	200	2	30	50	4.13	4.51	1.09	3.13	3.21	1.03	1.27	1.32	1.03	2.46
	200	2	30	100	8.76	9.14	1.04	4.78	4.99	1.04	1.73	1.78	1.03	2.76
Batch				12.5	1.45	1.69	1.17	1.89	1.99	1.05	0.93	0.98	1.06	2.05
				25	2.09	2.45	1.17	2.19	2.30	1.05	1.02	1.07	1.05	2.16
				50	3.87	4.38	1.13	3.08	3.21	1.04	1.38	1.45	1.05	2.23
				100	8.51	8.91	1.05	4.31	4.48	1.04	1.70	1.79	1.05	2.54

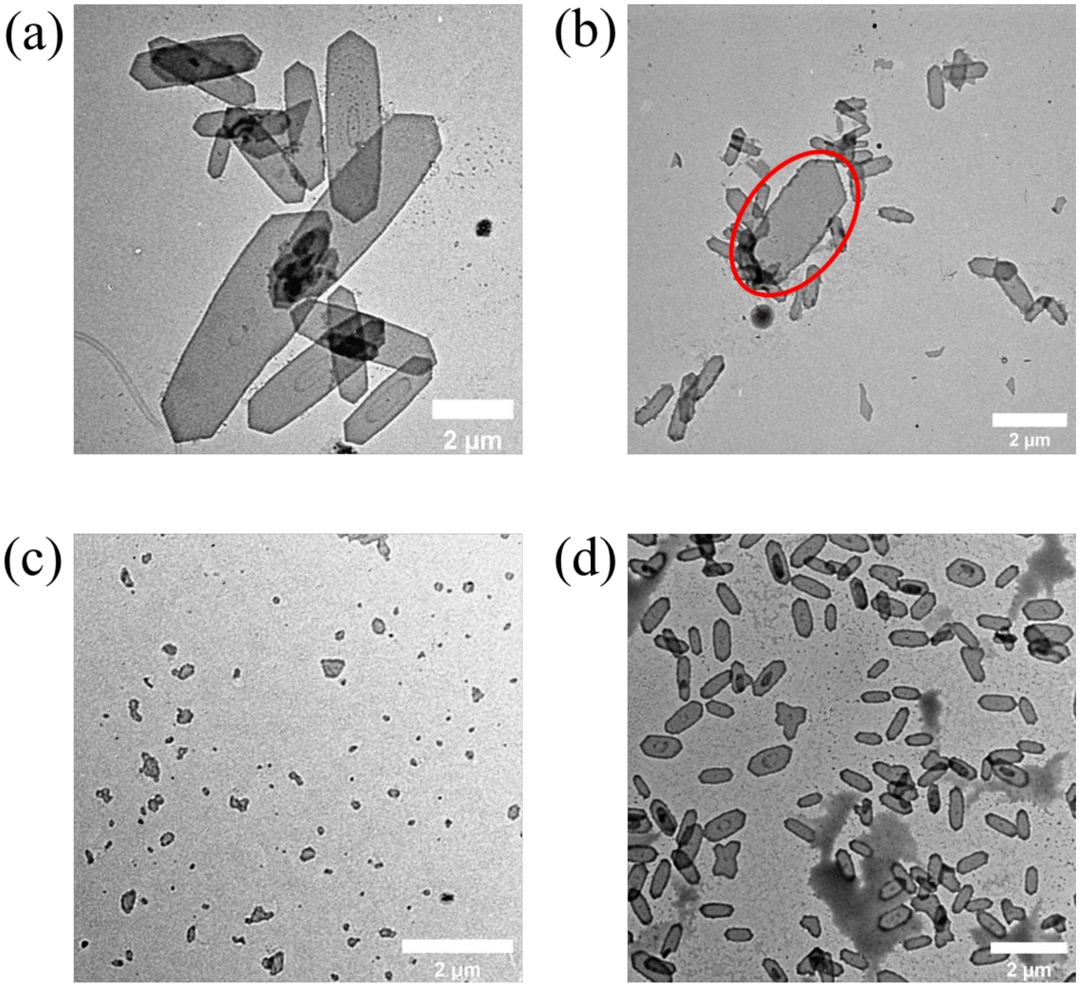

Figure S12. (a) TEM images of extended flow platelets obtained from wider tubing, (b) flow platelets from seeds at the unimer/seed ratio of 10, (c) fragments of platelets, and (d) flow platelets from platelet fragments at the unimer/seed ratio of 10.

Table S12. Flow platelets from seeds with various amount of unimer.

Unimer	Area (um^2)			Length (um)			Width (um)			
(eqv. of seed)	A_n	A_w	A_w/A_n	L_n	L_w	L_w/L_n	W_n	W_w	W_w/W_n	L/W
5	0.27	0.35	1.31	0.84	0.87	1.05	0.32	0.33	1.05	2.63
10	0.59	0.66	1.11	1.22	1.27	1.04	0.43	0.45	1.04	2.80
20	1.35	1.48	1.09	1.95	2.00	1.03	0.68	0.70	1.04	2.88
30	2.41	2.55	1.06	2.87	2.91	1.01	0.96	0.99	1.02	2.98

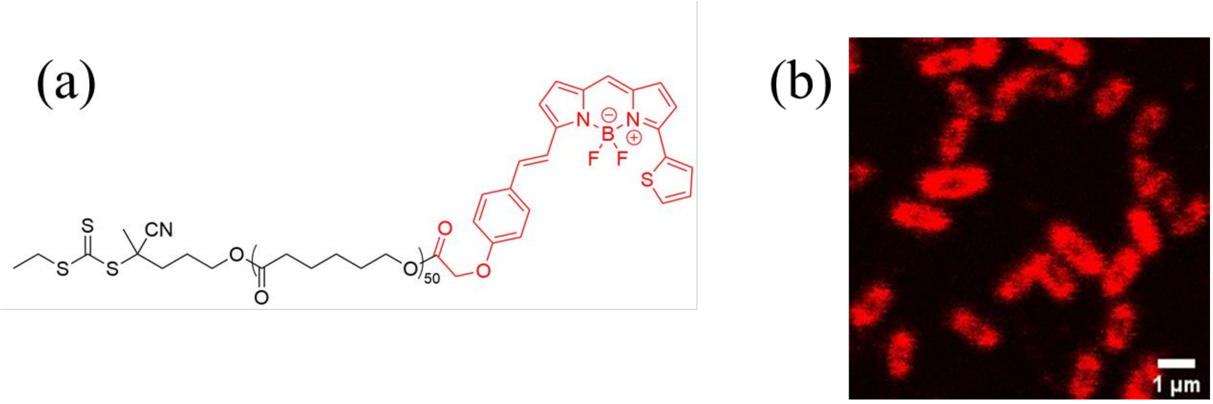

Figure S13. (a) molecular structure of bodipy 630/650 modified PCL and (b) confocal microscopy image (scale bar = 20 μm) of fluorescently labelled platelets.

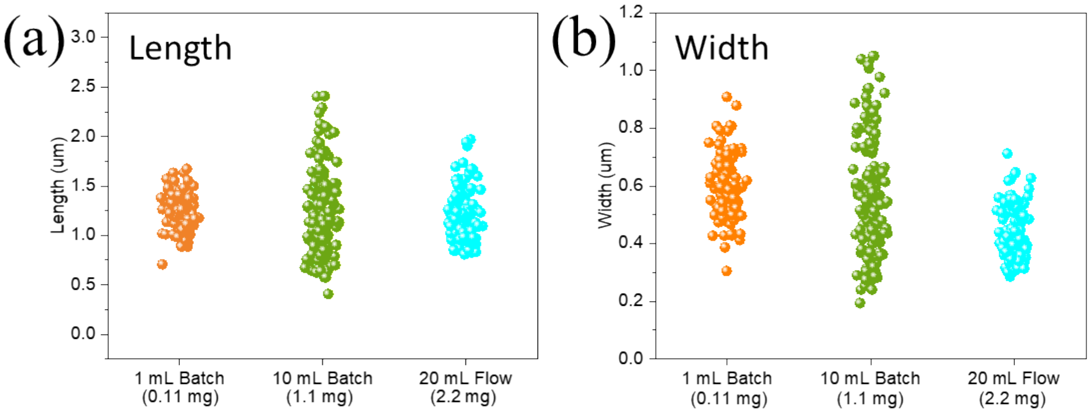

Figure S14. Size comparison of platelets from 1-mL and 10-mL batch scales and 20-mL flow: (a) length and (b) width.

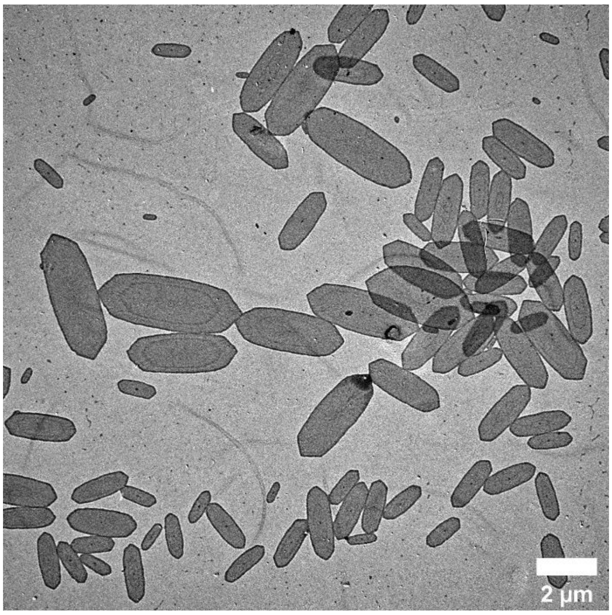

Figure S15. TEM image of platelets prepared at 1 mg mL^{-1} in flow reactors.
